# Mixed Aldosterone and Cortisol Secretion in Patients with Adrenal Incidentalomas: Different Faces of the Old Concept of Connshing Syndrome

**DOI:** 10.7759/cureus.92759

**Published:** 2025-09-19

**Authors:** Teodora Kamenova, Aneliya Nankova, Ivesta Kirova, Georgi Kirilov, Sabina Zacharieva, Atanaska Elenkova

**Affiliations:** 1 Endocrinology, University Hospital of Endocrinology, Medical University Sofia (USHATE) "Acad. Ivan Penchev", Sofia, BGR; 2 Pituitary Clinic, University Hospital of Endocrinology, Medical University Sofia (USHATE) "Acad. Ivan Penchev", Sofia, BGR

**Keywords:** co-secretion, excess cortisol, incidentalomas, laparoscopic adrenalectomy, primary aldosteronism

## Abstract

Background: Co-secretion of aldosterone and cortisol from an adrenal adenoma is considered a clinically significant condition that can negatively affect blood pressure (BP) control and metabolic status. Studies in cohorts with primary aldosteronism (PA) have identified autonomous cortisol secretion (ACS) in a significant proportion of these patients. However, the frequency and clinical characteristics of this so-called “Connshing syndrome” among patients with adrenal incidentalomas (AIs) remain not sufficiently evaluated.

Objective: The main aim of the study was to investigate, in a prospective design, the prevalence and the clinical characteristics of mixed aldosterone and cortisol secretion among patients with AIs referred to our expert center. According to our hypothesis, we expected a low prevalence and an atypical clinical presentation in these patients.

Methods: PA was diagnosed based on the aldosterone/renin ratio (ARR) as a screening test, followed by two confirmatory tests: the captopril challenge test (CCT) and the dexamethasone-captopril-valsartan test (DCVT). Unilateral aldosterone-cortisol oversecretion was confirmed by imaging studies (computed tomography (CT) or magnetic resonance imaging (MRI)) in all patients and adrenal venous sampling (AVS) in the single patient with bilateral adenomas. ACS was confirmed via the overnight 1 mg dexamethasone suppression test (DST).

Results: We conducted a prospective study on 399 patients with AIs referred to our expert center during the last three years (from May 10, 2022, to May 10, 2025). Aldosterone and cortisol co-secretion (A/C-CoS) was identified in four patients (≈1%), presenting different clinical manifestations. Three patients had well-controlled hypertension on conventional antihypertensive therapy, and one was normotensive. Three of the four patients had dyslipidemia. Carbohydrate disturbances were found in two patients with normal body mass index (BMI) (one woman with impaired fasting glucose and one man with overt diabetes mellitus (DM)). The other two subjects were slightly overweight with normal glucose tolerance.

Conclusion: Mixed aldosterone and cortisol hypersecretion is usually associated with poor BP control and metabolic disturbances, but in real clinical practice, a wide range of disorders is observed, from severe resistant hypertension to rare cases of normotension and from pronounced metabolic syndrome to a complete absence of metabolic abnormalities. Atypical presentation of A/C-CoS is more likely to be found among patients with AIs. This observation confirms the need for a comprehensive hormonal evaluation of all patients with AIs, regardless of their clinical presentation.

## Introduction

Aldosterone and cortisol co-secretion (A/C-CoS) was first reported in 1979 under the term “Connshing syndrome” [1]. In 2017, Arlt et al. renewed interest in the topic [2]. The data from their study showed that glucocorticoid co-secretion is commonly observed in primary aldosteronism (PA) and contributes to higher metabolic risk [2]. This mixed hormonal excess can lead to a variety of symptoms and conditions, including high blood pressure (BP), hypokalemia, increased risk of insulin resistance, higher body mass index (BMI), prediabetic states, or overt diabetes mellitus (DM) [3, 4]. Hypertension is a major concern due to its strong association with increased cardiovascular risk and mortality [4]. It is also clear that monotherapy with mineralocorticoid receptor antagonists is not sufficient to counteract the adverse metabolic risk [2]. The term "Connshing syndrome" is not entirely accurate today, because A/C-CoS can be observed not only in Conn's adenoma, but also in some patients with bilateral adrenal hyperplasia (BAH) [5]. 

Recently published meta-analyses have shown a relatively high prevalence of autonomous cortisol secretion (ACS) in patients with PA due to Conn's adenoma (up to 20% to 30%) [5]. On the other hand, the prevalence of Conn's adenoma among patients with adrenal incidentalomas (AIs) is very low. A previous retrospective study conducted in our expert center, including 515 patients with AIs, determined its prevalence to be approximately 1% [6]. Based on this data, we hypothesized that cases of A/C-CoS among patients with AIs are expected to be extremely rare and likely to present with diverse features, even without the specific signs and symptoms of PA and Cushing’s syndrome.

## Materials and methods

Study design

We conducted a prospective study among patients with AIs referred to the University Specialized Hospital for Active Treatment in Endocrinology (USHATE) "Acad. Ivan Penchev," Sofia, Bulgaria, during the last three years (from May 10, 2022, to May 10, 2025). All patients with AIs (n=399) who met the inclusion criteria and did not have any exclusion criteria were enrolled.

Aim of the study

The primary aim of this study was to investigate the prevalence, clinical presentation, and disease course in patients with A/C-CoS among a cohort of subjects with AIs. The secondary aim was to evaluate the outcome after adrenalectomy in patients with A/C-CoS. 

The study was approved by the Ethics Committee of the hospital (approval number: 001/2022). Written informed consent was obtained from all subjects at their admission to the hospital before any study procedure. The main inclusion criterion was adrenal adenoma visualized on imaging studies (computed tomography (CT) or magnetic resonance imaging (MRI)) performed prior to hospitalization for indications unrelated to adrenal pathology.

Exclusion criteria

The exclusion criteria were as follows: 1. Adrenal lesion discovered due to suspicion for endocrine hypertension or adrenal pathology; 2. Patients with confirmed oncological diseases (due to the risk that their adrenal lesions could be metastatic).

Materials and methods

A comprehensive hormonal evaluation was performed in all 399 patients included in the study. The diagnosis of PA was established in accordance with the recommendations of the European Society of Endocrinology [7]. The renin-angiotensin-aldosterone system (RAAS) was initially assessed by determining the aldosterone/renin ratio (ARR) as a screening test in all study participants after discontinuation of the intake of medications affecting RAAS (six weeks for mineralocorticoid receptor antagonists (MRA); two weeks for angiotensin receptor blockers (ARB), angiotensin-converting-enzyme (ACE) inhibitors, and thiazide and loop diuretics) [7]. Two confirmatory tests were used in cases with abnormal results of the screening test (ARR > 750 pmol/l/ng/ml/h): the captopril challenge test (CCT) and the two-day dexamethasone-captopril-valsartan test (DCVT). CCT was performed in all cases with abnormal results of the screening test (ARR > 750 pmol/l/ng/ml/h). In patients with A/C-CoS, the second confirmatory test (DCVT) was conducted.

The CCT was selected because of its low risk of adverse reactions and complications and easy implementation [7]. The levels of plasma renin activity (PRA) and aldosterone were measured at baseline (after 30 minutes of rest) and at the 90^th^ minute following oral administration of 50 mg of captopril in the seated position. Radioimmunoassays (RIA) were used for aldosterone and angiotensin I (IMMUNOTECH, Beckman Coulter, Prague, Czech Republic) detection. Angiotensin I was used for the quantitative measurement of PRA.

The DCVT was performed according to the protocol established by the authors who introduced the test [8]. On Day 1, at midnight, 2 mg dexamethasone, 50 mg captopril, and 320 mg valsartan were administered orally. On Day 2, at 7 AM, the second dose of 50 mg captopril was given. At 8 AM, blood sampling for adrenocorticotropic hormone (ACTH), PRA, cortisol, and aldosterone was done. All blood samples were drawn after at least 30 minutes of rest in a sitting position. We selected this second confirmatory test in cases with A/C-CoS due to its dual pharmacologic blockade on both the main physiological stimulators of aldosterone and cortisol secretion, angiotensin II and ACTH [8].

Cortisol secretion was evaluated by measuring ACTH, 24-hour urinary free cortisol excretion (UFC), and conducting the 1 mg overnight dexamethasone suppression test (DST). ACS was confirmed by DST using the cut-off value of > 50 nmol/L (>1.8 µg/dL) for second-day cortisol at 8.00 AM after taking 1 mg dexamethasone at midnight [9]. The 24-hour UFC (nmol/24 h), as well as the serum cortisol, was measured by highly sensitive and specific RIA (Immunotech, Beckman Coulter Co., France). Intra-assay and inter-assay coefficients of variations were for serum ≤ 5.8 % and 9.2 %, respectively; for urine samples ≤ 8.9 % and 13.3 %, respectively; and for urine extracts ≤ 9.4 % and 12.6 %, respectively. Analytical sensitivity was 5 nM. Extremely low cross-reactivities were obtained against other naturally occurring steroids (aldosterone, corticosterone, cortisone, 11-deoxycorticosterone (11-DOC), progesterone, etc.) or therapeutic drugs (prednisolone, prednisone, spironolactone, etc.) after dichloromethane extraction of the urine samples before testing. Plasma ACTH was determined by the highly sensitive, specific ImmunoRadiometric Assay (IRMA) method (ACTH Thermo Scientific BRAHMS, Hennigsdorf, Germany) with analytical sensitivity of 0.26 pmol/l and functional sensitivity, measured by 20% intertest variation coefficient, of 0.52 pmol/l. Dehydroepiandrosterone sulfate (DHEA-S) was also measured in all study participants.

Adrenomedullary function was assessed by measuring 24-hour urine fractionated metanephrines (metanephrine and normetanephrine). Thyroid function was evaluated by assessing thyroid-stimulating hormone (TSH) and free thyroxine (fT4).

To determine lateralization of the process, adrenal vein sampling (AVS) was performed in one patient with bilateral adrenal adenomas. The procedure was performed after discontinuation of antihypertensive medications with potential impact on the RAAS (ACE inhibitors, ARBs, MRAs, and diuretics).

Statistical analysis

Descriptive statistics were used for demographic and laboratory data. The frequencies of the adrenal tumor types were presented as percentages. The Shapiro-Wilk test was applied to check the normality of the sample distribution. Metric variables were described by the number of observations (n), arithmetic mean ± standard deviation (SD) in samples with normal (Gaussian) distribution, or median and interquartile range (IQR) when data distribution was not normal.

## Results

Only four of the 399 patients investigated (1%) with confirmed A/C-CoS were identified, presenting a wide range of cardiometabolic disorders. The largest proportion of the studied cohort (67.7%), as expected, consisted of patients with non-functioning adrenal tumors. Mild ACS represented the second most common subgroup of patients identified (26.55%), followed by pheochromocytomas (3%) and PA (1.75%), identified as AIs. The main demographic characteristics and laboratory parameters of the entire cohort are presented in Table 1. Comparative analysis between the groups was not conducted due to substantial disparities in sample sizes and the insufficient number of observations in three of the groups.

**Table 1:** Main parameters of all the study participants The data are presented as mean ± SD or median (IQR), depending on the sample distribution. NFA: non-functioning adrenal tumors; MACS: mild autonomous cortisol secretion; APA: aldosterone producing adenoma; A/C-CoS: mixed aldosterone/cortisol secretion; Pheo: pheochromocytoma; n: number of patients; SD: standard deviation; LDL-cholesterol: low-density lipoprotein cholesterol; HDL-cholesterol: high-density lipoprotein cholesterol; eGFR (CKD-EPI): estimated glomerular filtration rate using the chronic kidney disease epidemiology collaboration equation; TSH: thyroid-stimulating hormone; UFC: urinary free cortisol; DST: dexamethasone suppression test; ACTH: adrenocorticotropic hormone; DHEA-S: dehydroepiandrosterone-sulfate; CCT: captopril challenge test; PRA: plasma renin activity; ARR: aldosterone/renin ratio *For descriptive purposes, all values of PRA below the limit of detection (LOD=0.3) were imputed as 0.15 (LOD/2). **All PRA values are below the LOD.

Parameter (units)	NFA (n=270)	MACS (n=106)	APA (n=7)	A/C-CoS (n=4)	Pheo (n=12)	Normal range
Percentage (%) of the cohort (female/male)	67.7% (204/66)	26.55% (76/30)	1.75% (3/4)	1.0% (3/1)	3.0% (7/5)	NA
Age (years)	59.5 ± 11.5	60.8 ± 11.1	57.0 ± 10.0	58.7 ± 6.65	58.6 ± 9.95	NA
Maximum tumor size (mm) median (min-max)	22.0 (8.0 – 120)	25.1 (8.0 – 60.0)	16.6 (12.6 – 25.0)	33.5 (18.0 – 46.0)	26.0 (10.0 – 59.0)	NA
Glucose (fasting morning) (mmol/l)	5.87 ± 1.26	5.73 ± 1.30	6.08 ± 0.68	5.73 ± 0.80	7.22 ± 2.46	3.89 – 6.1
Creatinine (µmol/l)	70.5 ± 21.2	71.7 ± 23.1	76.4 ± 18.5	62.2 ± 8.17	70.2 ± 16.5	44 – 85
Total cholesterol (mmol/l)	5.23 ± 1.21	5.21 ± 1.32	5.47 ± 0.73	5.39 ± 1.65	5.34 ± 1.15	< 5.2
HDL-cholesterol (mmol/l)	1.43 ± 0.36	1.43 ± 0.41	1.39 ± 0.41	1.47 ± 0.18	1.30 ± 0.19	> 1.55
Triglycerides (mmol/l)	1.45 ± 0.71	1.40 ± 0.75	1.79 ± 1.03	1.07 ± 0.58	1.75 ± 0.72	< 1.70
Uric acid (µmol/l)	339.8 ± 98.9	332.3 ± 87.7	321.6 ± 65.5	296.0 ± 25.6	332.3 ± 91.9	142 – 416
Potassium (mmol/l)	4.61 ± 0.46	4.47 ± 0.64	3.96 ± 0.49	4.43 ± 0.38	4.58 ± 0.52	3.5 – 5.6
TSH (mmol/l)	2.84 ± 1.53	2.39 ± 1.61	2.84 ± 1.53	1.44 ± 0.81	1.96 ± 0.76	0.3 – 4.2
UFC (nmol/l)	133.7 ± 95.2	165.6 ± 131.0	106.8 ± 56.1	177.0 ± 158	153.0 ± 96.1	< 208
Cortisol after 1 mg DST (nmol/l)	46.3 ± 17.6	147.2 ± 99.6	33.53 ± 6.42	186.4 ± 95.6	59.2 ± 28.7	<50
ACTH (pmol/l)	7.32 ± 4.20	5.55 ± 3.93	7.55 ± 6.74	7.55 ± 6.74	6.5 ± 5.05	1.6 – 13.9
DHEA-S (µmol/l)	2.86 ± 2.30	2.33 ± 2.26	2.48 ± 1.51	2.95 ± 2.90	4.40 ± 2.42	0.9 – 9.0
Aldosterone (baseline) (pmol/l)	228.1 [213.5]	220.5 [166.5]	1142.9 ± 701.9	729.2 ± 305.7	156.0 [103.8]	116 – 580
PRA (baseline) (ng/ml/h)	1.52 ± 4.31	0.90 ± 1.33	0.18 ± 0.08*	0.15 ± 0.0**	0.82 ± 0.42	0.32 -1.84
ARR (pmol/l/ng/ml/h)	358.0 [510.0]	560 [441.0]	7229.0 ± 5063.2	4859.9 ± 2039.1	237.2 ± 139.8	<750
Metanephrine (urine) (ng/24h)	85.5 [76.3]	78.0 [82.1]	127.3 [187.6]	146.8 ± 102.8	206.5 [283.8]	<350
Normetanephrine (urine) (ng/24h)	436.0 [312.0]	398 [304.0]	336.5 [381.2]	317.1 ± 209.6	2127 [1790.8]	<650

Given the main focus of the study and the small number of patients with A/C-CoS, we provide a brief presentation of each clinical case in chronological order.

Patient 1

**Figure 1:**
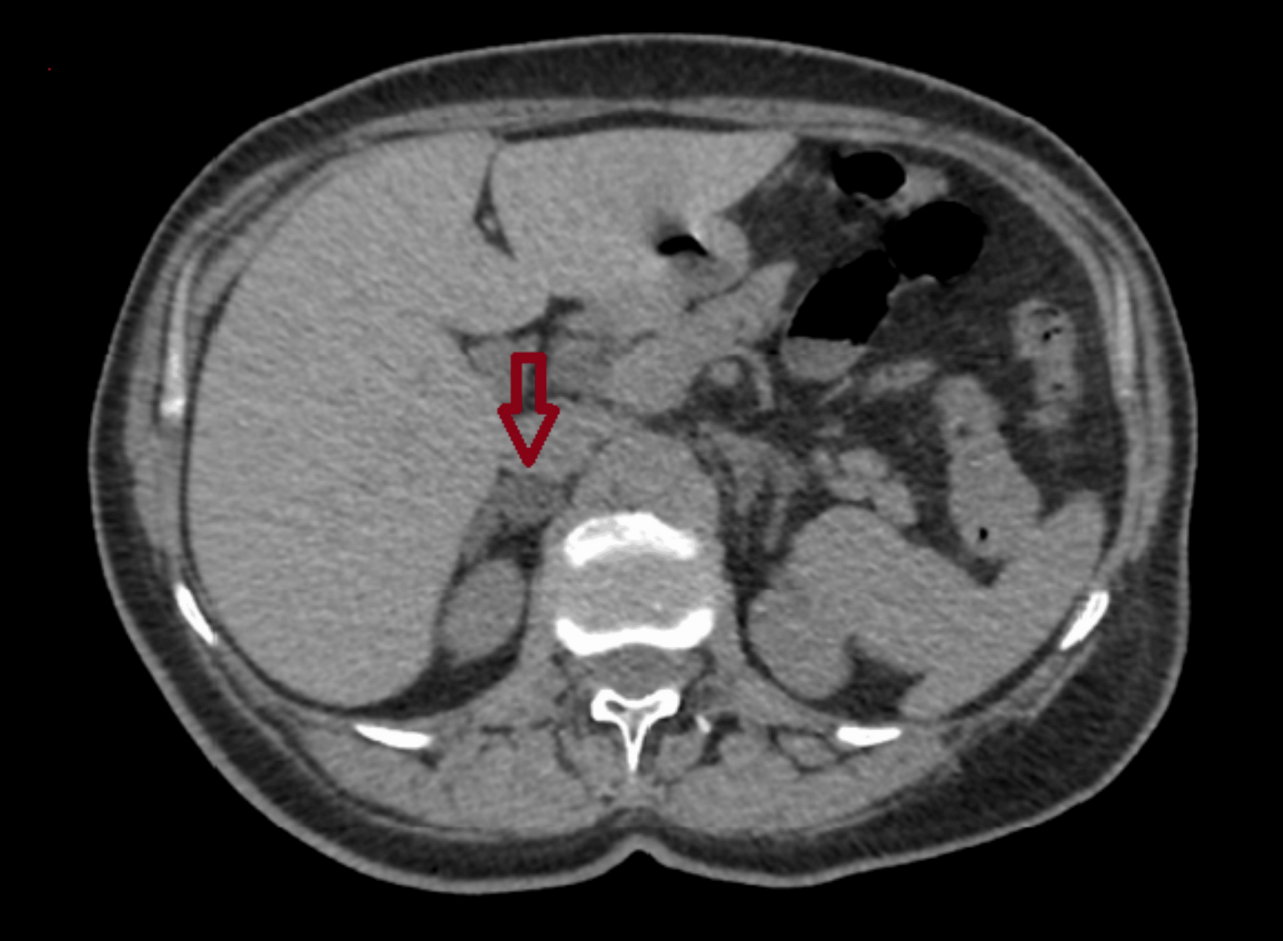
Adrenal imaging findings of Patient 1 An abdominal CT (transverse plane) of a 67-year-old woman showing a right adrenal incidentaloma measuring 18 × 15 mm.

A 67-year-old woman was admitted to the clinic in May 2022 for hormonal evaluation of an incidentaloma of the right adrenal gland, sized 18x15 mm (Figure 1). The patient had a 20-year history of hypertension treated with an ARB and a beta-blocker. Peak BP values reached 240/120 mmHg on a single occasion. Overall, arterial hypertension (AH) was well controlled. Several years ago, due to unexplained progressive weight gain, the patient was referred to an endocrinologist. Autoimmune hypothyroidism was found, and a levothyroxine substitution therapy was started. Over the past few years, she lost 29 kg of her weight as a result of levothyroxine therapy and a diet and exercise regimen. A stable euthyroid state was achieved. The patient reported a total hysterectomy with bilateral adnexectomy for uterine myomatosis and right ovary cystadenofibroma. Her family history was positive for AH. The core anthropometric measurements during the physical examination were as follows: height: 160 cm; weight: 68 kg; waist circumference: 91 cm; and BMI: 26.5 kg/m². The main vital signs were BP: 120/80 mmHg; rhythmic normofrequent heart rate (HR) of 75 beats per minute (bpm). Main laboratory parameters at diagnosis are presented in Table 2. A study of the RAAS was conducted. Diagnostic tests revealed a high ARR, increased aldosterone, and persistently suppressed PRA during both CCT and DCVT (Table 3). There was also an elevated midnight serum cortisol level and non-suppression after 1 mg DST. Hypercatecholaminemia was excluded. Results were consistent with adrenal-related synchronous aldosterone and cortisol excess. Normal glucose tolerance was confirmed, and dyslipidemia was found. The patient refused surgical treatment. We recommended therapy with a calcium antagonist, an ARB, an alpha-adrenergic agonist, and a beta-blocker, as well as antilipemic treatment and a regular follow-up at the clinic. However, the patient refused to visit the clinic for further testing. During the periodic telephone calls, she reported well-controlled hypertension and normokalaemia with regular intake of the above-mentioned antihypertensive medications in the dosage regimen prescribed during her first hospitalization. 

**Table 2:** Laboratory results of the patients at diagnosis: biochemistry eGFR (CKD-EPI): estimated glomerular filtration rate using the chronic kidney disease epidemiology collaboration equation; HDL-cholesterol: high-density lipoprotein cholesterol; LDL-cholesterol: low-density lipoprotein cholesterol

Parameter (units)	Patient 1	Patient 2	Patient 3	Patient 4	Normal range
Glucose (mmol/l)	4.8	5.82	6.74	5.59	3.89 – 6.1
Creatinine (µmol/l)	58	53	66.6	71.03	44 – 85
eGFR (CKD-EPI) (ml/min/1,73m^2^)	96.2	107.2	103.2	86	> 90
Total cholesterol (mmol/l)	6.03	3.99	4.11	7.44	< 5.2
HDL-cholesterol (mmol/l)	1.46	1.67	1.24	1.5	> 1.55
LDL-cholesterol (mmol/l)	4.15	2.34	2.69	5.53	< 2.59
Triglycerides (mmol/l)	1.37	0.54	0.63	1.76	< 1.70
Uric acid (µmol/l)	297	322	304	261	142 – 416
Sodium (mmol/l)	146	145	148	149	136 – 151
Potassium (mmol/l)	4.8	2.7	4.1	4.7	3.5 – 5.6
Chloride (mmol/l)	106	98	105	105	96 – 101

**Table 3:** Laboratory results of the patients at diagnosis: hormonal analysis and dynamic tests TSH: thyroid-stimulating hormone; UFC: urinary free cortisol; DST: dexamethasone suppression test; ACTH: adrenocorticotropic hormone; DHEA-S: dehydroepiandrosterone sulfate; CCT: captopril challenge test; PRA: plasma renin activity; ARR: aldosterone/renin ratio; DCVT: dexamethasone-captopril-valsartan test

Parameter (units)	Patient 1	Patient 2	Patient 3	Patient 4	Normal range
TSH (mmol/l)	0.48	1.5	1.33	2.46	0.3 – 4.2
UFC (nmol/l)	100.5	172.7	399.8	35	< 208
Cortisol after 1 mg DST (nmol/l)	150.2	233.7	71.8	290	<50
ACTH (pmol/l)	3.1	2.03	6.77	0.715	1.6 – 13.9
DHEA-S (µmol/l)	NA	2.4	6.11	0.35	0.9 – 9.0
Aldosterone 0` (pmol/l)	449	1165	653	649	116 – 580
Aldosterone 90` after CCT (pmol/l)	621	382	442	912	<330
PRA 0` (ng/ml/h)	<0.3	<0.3	<0.3	<0.3	0.32 -1.84
PRA 90` after CCT (ng/ml/h)	<0.3	<0.3	<0.3	<0.3	NA
ARR (pmol/l/ng/ml/h)	2993.3	7766.7	4353.3	4326.6	<750
Aldosterone after DCVT (pmol/l)	298	718	271	727	<85
PRA after DCVT (ng/ml/h)	<0.3	0.5	0.5	<0.3	>0.3
Cortisol after DCVT (nmol/l)	199.9	64.5	48.5	275	NA
ACTH after DCVT (pmol/l)	3.1	0.572	0.456	<0.33	NA
ARR after DCVT (pmol/l/ng/ml/h)	1986.7	1436	542	4864.7	<9
Metanephrine (urine) (ng/24h)	66	50	216.6	67.8	<350
Normetanephrine (urine) (ng/24h)	271	154.3	556.8	173.1	<650

Patient 2

**Figure 2:**
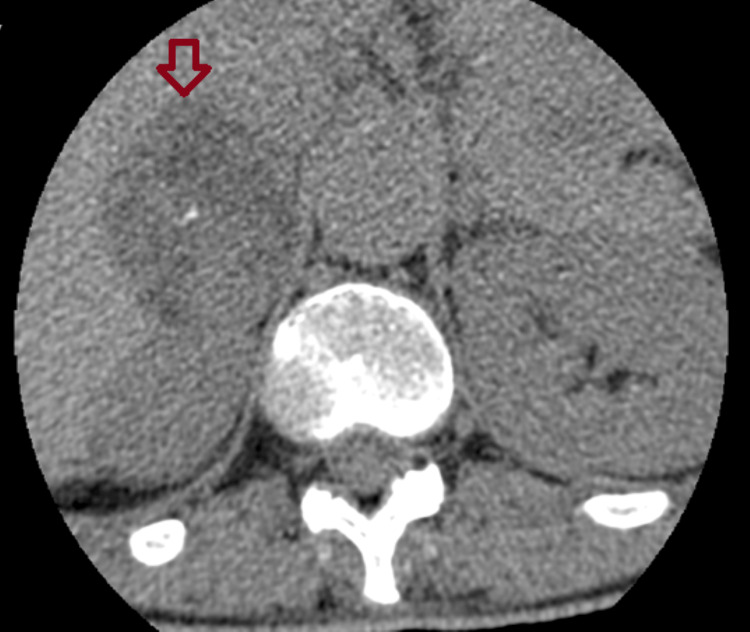
Adrenal imaging findings of Patient 2 An abdominal CT (transverse plane) of a 51-year-old woman showing a right adrenal incidentaloma measuring 46 × 43 mm.

A 51-year-old woman with a right adrenal gland incidentaloma (size 46x43 mm) was admitted to our expert center in October 2022. The lesion was discovered on a CT scan due to lumbalgia (Figure 2). The patient reported AH from the age of 41, well-controlled at admission on a four-drug combination antihypertensive therapy (ACE inhibitor, calcium antagonist, imidazoline receptor antagonist, and a beta-blocker). She reported impaired fasting glycemia (IFG). Her past medical history included partial thyroid resection due to a benign thyroid nodule and a supraventricular ablation due to paroxysmal tachycardia. She had a family history of DM and AH. The main findings from the physical examination were as follows: height: 160 cm, weight: 62 kg, waist circumference: 86 cm, and BMI: 24.2 kg/m². BP was 120/80 mmHg, and HR was 78 bpm. Laboratory examination revealed hypokalemia, high ARR and aldosterone levels, and suppressed PRA. There was no suppression of cortisol level after 1 mg DST. Results were consistent with adrenal-related synchronous aldosterone and cortisol excess. The patient was referred to the surgical department, and a right-sided laparoscopic adrenalectomy was performed. The postoperative follow-up, three months after laparoscopic adrenalectomy, demonstrated normalization of serum potassium levels, low serum aldosterone, still suppressed PRA, and plasma ACTH. AH was well-controlled on the background of triple antihypertensive therapy (ARB, diuretics, and beta-blockers). At the follow-up visit 18 months after surgery, AH was still well-controlled on the same triple antihypertensive therapy, but with the beta-blocker dose reduced by half (Table 4).

**Table 4:** Patient 2's laboratory results before and after laparoscopic adrenalectomy eGFR (CKD-EPI): estimated glomerular filtration rate using the chronic kidney disease epidemiology collaboration equation; HDL-cholesterol: high-density lipoprotein cholesterol; LDL-cholesterol: low-density lipoprotein cholesterol; TSH: thyroid-stimulating hormone; UFC: urinary free cortisol; DST: dexamethasone suppression test; ACTH: adrenocorticotropic hormone; DHEA-S: dehydroepiandrosterone-sulfate; CCT: captopril challenge test; PRA: plasma renin activity; ARR: aldosterone/renin ratio; DCVT: dexamethasone-captopril-valsartan test

Laboratory data	Before surgery	3 months after surgery	18 months after surgery	Normal range
Glucose (mmol/l)	5.82	5.3	6.63	3.89 – 6.1
Creatinine (µmol/l)	53	62	61.51	44 – 85
eGFR (CKD-EPI) (ml/min/1,73m^2^)	107.2	104.4	104.1	>90
Total cholesterol (mmol/l)	3.99	4.0	4.45	<5.2
HDL-cholesterol (mmol/l)	1.67	1.4	1.76	> 1.0
LDL-cholesterol (mmol/l)	2.34	2.2	2.66	< 2.59
Triglycerides (mmol/l)	0.54	0.74	0.69	< 1.72
Uric acid (µmol/l)	322	329	420	142 – 416
Sodium (mmol/l)	145	148	144	136 – 151
Potassium (mmol/l)	2.7	4.8	4.9	3.5 – 5.6
Chloride (mmol/l)	98	101	101	96 – 101
TSH (mmol/l)	1.5	0.76	0.58	0.3 – 4.2
UFC (nmol/24h)	172.7	NA	NA	< 208
Cortisol after 1 mg DXM (nmol/l)	233.7	55.1	42	<50
АCTH (pmol/l)	2.03	7.98	NA	2.2 – 12.2
DHEA-S (umol/l)	2.4	NA	NA	3.6 – 12
Aldosterone 0` (pmol/l)	1165	90	81	55 – 470
Aldosterone 90` after CCT (pmol/l)	382	<76	NA	<330
PRA 0` (ng/ml/h)	<0.3	<0.3	14.6	0.3 -3.5
PRA 90` after CCT (ng/ml/h)	<0.3	<0.3	NA	NA
ARR (pmol/l/ng/ml/h)	7766.7	600	5.58	<750
Aldosterone after DCVT (pmol/l)	718	76	NA	<85
PRA after DCVT (ng/ml/h)	0.5	0.7	NA	>0.3
Cortisol after DCVT (nmol/l)	NA	64.5	NA	NA
ACTH after DCVT (pmol/l)	0.337	0.572	NA	NA
ARR after DCVT (pmol/l/ng/ml/h)	1436	108.57	NA	< 9
Metanephrine (urine) (ng/24h)	50	NA	153.8	< 350
Normetanephrine (urine) (ng/24h)	154.3	NA	554.6	< 650

Patient 3

**Figure 3:**
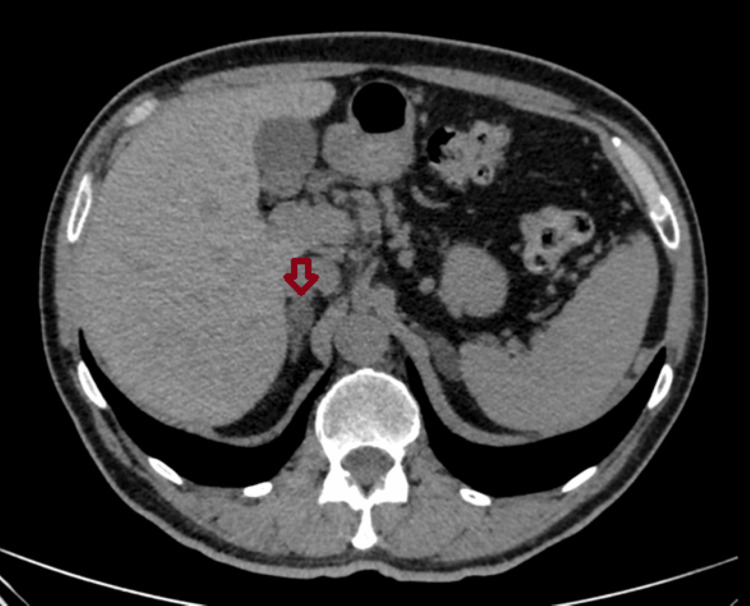
Adrenal imaging findings of Patient 3 An abdominal CT (transverse plane) of a 60-year-old man with bilateral adrenal incidentalomas (an incidentaloma in the right adrenal gland measuring 15×14 mm).

**Figure 4:**
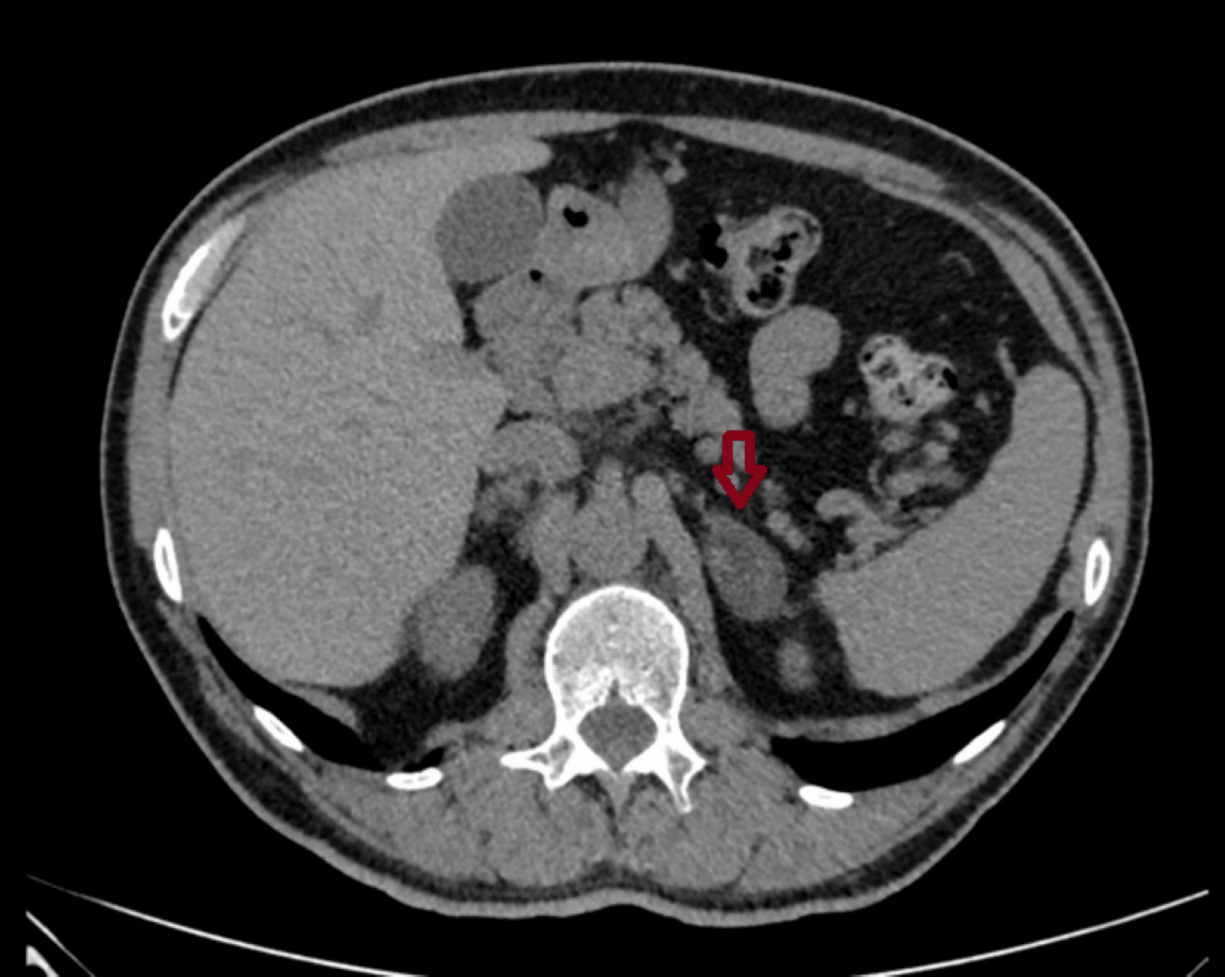
Adrenal imaging findings of Patient 3 An abdominal CT (transverse plane) of a 60-year-old man with bilateral adrenal incidentalomas (an incidentaloma in the left adrenal gland measuring 37×22 mm).

A 60-year-old man with bilateral AIs (right 15x14 mm; left 37x22 mm), detected on CT due to nephrolithiasis, was referred to our expert center for hormonal evaluation. The lesions were described as hypodense with an inhomogeneous increase in density during the arterial phase after contrast application (Figures 3, 4). In addition, a left kidney intraparenchymal lipoma and peripelvic cysts were also visualized. The patient reported a several-year history of DM. The patient reported a several-year history of DM with good glycemic control (HbA1c: 5.9%) on treatment with metformin and a dipeptidyl peptidase-4 (DPP-4) inhibitor. Another concomitant condition was left anterior fascicular block and right bundle branch block. His BP was normal without antihypertensive therapy. Physical examination revealed the following: height: 186 cm; weight: 84 kg; waist circumference: 95 cm; BMI: 24.3 kg/m²; BP: 130/70 mmHg; and HR: 80 bpm. The RAAS examination revealed increased ARR, inadequate aldosterone suppression after the CCT, and persistently suppressed PRA. In addition, there was an increased 24-hour UFC and no suppression of cortisol levels after the 1 mg DST (Table 5). Due to the bilateral nature of AIs, the patient was referred for AVS, which showed lateralization of the hormonal overproduction and confirmed adrenal-related synchronous aldosterone and cortisol excess by the left AI (Table 6). A preoperative therapy with spironolactone and doxazosin was prescribed, and a left-sided laparoscopic adrenalectomy was performed in February 2025. The histological examination confirmed an adrenocortical adenoma. The immunohistochemistry showed positive expression of melan A and negative expression of chromogranin A. Postoperative follow-up three months after surgery revealed normalization of 24-hour urinary cortisol excretion, baseline aldosterone levels, and ARR; normal response during the CCT, but still suppressed PRA and lack of sufficient aldosterone suppression after DCVT (Table 5). BP remained within the normal range without treatment. Postoperative HbA1c levels corresponded to excellent glycemic control on the same antidiabetic therapy and dietary and physical activity regimen.

**Table 5:** Patient 3's laboratory results before and after laparoscopic adrenalectomy eGFR (CKD-EPI): estimated glomerular filtration rate using the chronic kidney disease epidemiology collaboration equation; HDL-cholesterol: high-density lipoprotein cholesterol; LDL-cholesterol: low-density lipoprotein cholesterol; TSH: thyroid-stimulating hormone; UFC: urinary free cortisol; DST: dexamethasone suppression test; ACTH: adrenocorticotropic hormone; DHEA-S: dehydroepiandrosterone-sulfate; CCT: captopril challenge test; PRA: plasma renin activity; ARR: aldosterone/renin ratio; DCVT: dexamethasone-captopril-valsartan test.

Laboratory data	Before surgery	Three months after surgery	Normal range
Glucose (mmol/l)	6.74	6.88	3.89 – 6.1
Creatinine (µmol/l)	66.6	79.99	44 – 85
eGFR (CKD-EPI) (ml/min/1,73m^2^)	103.2	97.1	> 90
Total cholesterol (mmol/l)	4.11	3.90	< 5.2
HDL-cholesterol (mmol/l)	2.69	1.11	> 1.55
LDL-cholesterol (mmol/l)	2.69	2.45	< 2.59
Triglycerides (mmol/l)	0.63	0.87	< 1.72
Uric acid (µmol/l)	304	366	142 – 416
Sodium (mmol/l)	148	147	136 – 151
Potassium (mmol/l)	4.1	4.8	3.5 – 5.6
Chloride (mmol/l)	105	108	96 – 101
TSH (mmol/l)	1.33	NA	0.3 – 4.2
UFC (nmol/24h)	399.8	117	< 208
Cortisol after 1 mg DST (nmol/l)	71.8	NA	<50
ACTH (pmol/l)	6.77	9.23	1.6 – 13.9
DHEA-S (µmol/l)	6.11	7.36	3.6 – 12
Aldosterone 0` (pmol/l)	653	217	116 – 580
Aldosterone 90` after CCT (pmol/l)	442	237	< 330
PRA 0` (ng/ml/h)	< 0.3	< 0.3	0.32 -1.84
PRA 90` after CCT (ng/ml/h)	< 0.3	< 0.3	NA
ARR (pmol/l/ng/ml/h)	4353.3	1446.7	< 750
Aldosterone after DCVT (pmol/l)	271	252	< 85
PRA after DCVT (ng/ml/h)	0.5	1.5	>0.3
Cortisol after DCVT (nmol/l)	48.5	< 40	NA
ACTH after DCVT (pmol/l)	0.456	0.417	NA
ARR after DCVT (pmol/l/ng/ml/h)	542	168	< 9
Metanephrine (urine) (ng/24h)	216.6	NA	< 350
Normetanephrine (urine) (ng/24h)	556.8	NA	< 650

**Table 6:** Patient 3's adrenal vein sampling results

Side	Cortisol (nmol/l)	Aldosterone (pmol/l)
Cubital vein	191	600
Left adrenal vein	5062	>6241
Right adrenal vein	241	505

Patient 4

**Figure 5:**
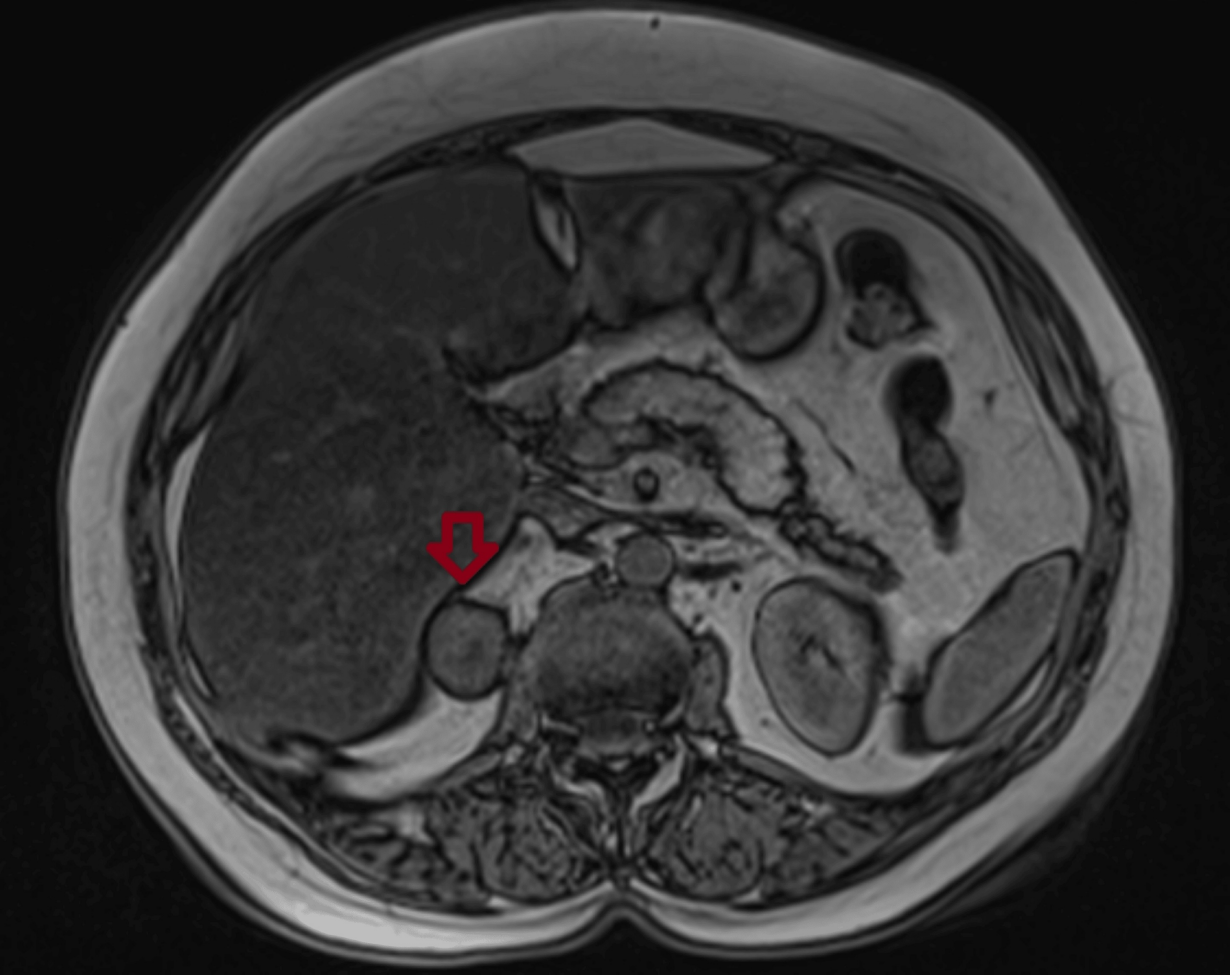
Adrenal imaging findings of Patient 4 An abdominal MRI (transverse plane) of a 57-year-old woman with a right adrenal incidentaloma measuring 30 mm.

**Figure 6:**
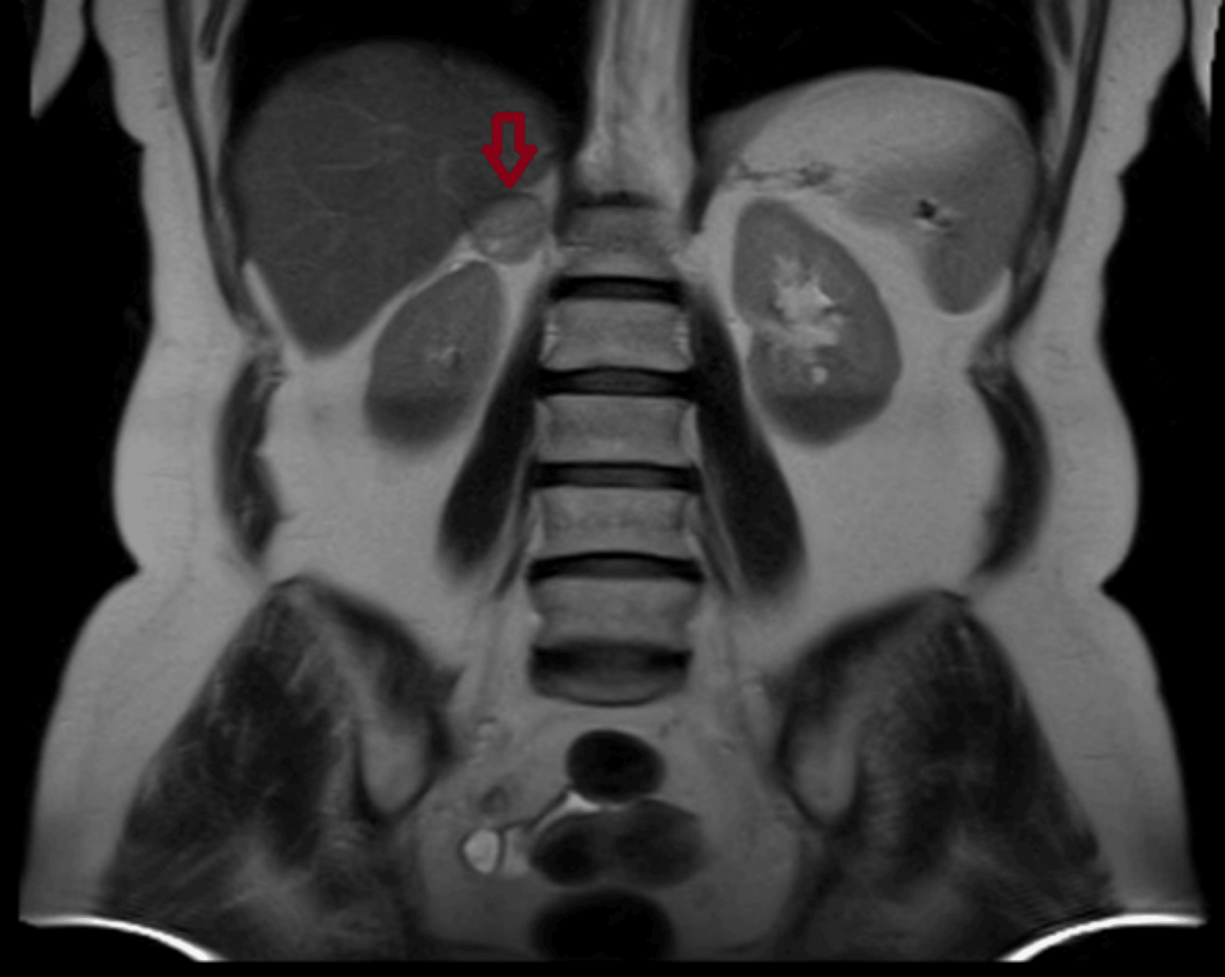
Adrenal imaging findings of Patient 4 An abdominal MRI (coronal plane) of a 57-year-old woman with a right adrenal incidentaloma measuring 30 mm.

A 57-year-old woman was admitted to the clinic in May 2025 for hormonal evaluation of an AI identified on MRI, which was performed due to right hypochondrial pain. A 30 mm mass was visualized in the right adrenal gland, described as clearly demarcated, fat-free, with the presence of small cystic components. There were no pathological findings in the left adrenal gland (Figure 5, Figure 6). In addition, a 10 mm lesion was found in the left kidney with imaging features suspicious of malignancy. The patient had AH, managed with triple antihypertensive therapy comprising a calcium antagonist, an ARB, and a diuretic. Despite this therapy, the BP ​​remained elevated over 140/90 mmHg with hypertensive crises accompanied by dizziness and nausea. The patient had gained 14 kg over the past three years. Dyslipidemia and normal glucose tolerance were confirmed (Table 2). Her past medical history included uterine myoma, right kidney angiomyolipoma, breast fibroadenoma, and right arm basocellular carcinoma. Her family history was notable for malignant diseases in both first- and second-degree relatives. Physical examination revealed an overweight individual with visceral fat distribution (height: 164 cm, weight: 74 kg, waist circumference: 102 cm, BMI=27.5 kg/m²), multiple nevi, arterial BP: 140/80 mm Hg, and a regular cardiac rhythm (HR: 65 bpm). Thyroid ultrasound visualized a suspicious nodule in the left thyroid lobe and an ipsilateral cervical lymph node with an altered structure. Diagnostic tests (Table 3) demonstrated high aldosterone levels with persistently suppressed PRA both at baseline and after CCT and DCVT, low ACTH levels, and elevated second-day serum cortisol after 1 mg DST. Results were consistent with adrenal-related synchronous aldosterone and cortisol excess. Preoperatively, spironolactone (50 mg daily) was added to the above-mentioned triple antihypertensive therapy. In June 2025, a robotic-assisted right adrenalectomy and a partial left renal resection were performed. Histological examination of the adrenal adenoma showed the presence of two cell populations: one composed of lipid-rich cells and the other composed of compact cells, forming solid nests and trabeculae. There were no histological signs of malignancy. The histological result of the renal tumor confirmed angiomyolipoma. Postoperatively, antihypertensive therapy was discontinued due to complete normalization of BP. Postoperative follow-up with a comprehensive hormonal evaluation is scheduled for September 2025. Fine-needle aspiration biopsy (FNAB) of the suspicious thyroid nodule will also be scheduled. 

## Discussion

Increasing evidence confirms that a subset of patients with adrenal adenomas exhibit A/C-CoS, which is considered to contribute to more severe AH, carbohydrate disturbances, dyslipidemia, obesity, multi-organ damage, and increased cardiovascular and cerebrovascular morbidity and mortality [10,11]. According to most published studies, the prevalence of ACS among patients with PA varies between 20% and 30%, depending on the diagnostic tests [5, 12]. On the other hand, the prevalence of Conn's adenoma among patients with AIs is quite low. A retrospective study conducted in our expert center on 515 Bulgarian patients with AIs determined its prevalence to be approximately 1% [6]. Based on these data, we hypothesized that cases of A/C-CoS among patients with AIs are extremely rare and likely present with atypical features: normokalemia, mild hypertension, or even normotension. We conducted this prospective study, which comprised 399 patients with AIs referred to our expert center for hormonal evaluation during the last three years. Only four patients with A/C-CoS were identified among this cohort. None of them presented with clinical features of an overt Cushing’s syndrome. This finding corresponds well with the literature data; most patients diagnosed with mixed A/C-CoS are characterized by subclinical hypercortisolism or mild ACS (MACS) [12,13]. In a comprehensive review of published studies on the topic, Spath et al. have recommended active screening of A/C-CoS among patients with PA with adenomas larger than 2.5 cm, a lack of cortisol suppression after 1 mg DST, and elevated serum levels of hybrid steroids [14]. Accordingly, three of our four patients had large adenomas, with maximum diameters of 30 mm, 37 mm, and 46 mm, respectively, but Patient 1 had a relatively small tumor (18 mm). There seems to be no correlation between the size of the adenoma and cortisol levels after DST, but the small number of patients does not allow for statistical analysis to test this hypothesis. According to recently published new guidelines of the Endocrine Society, DST is suggested for all patients with PA and adrenal adenoma [15]. 

PA is usually suspected in patients with resistant AH with or without spontaneous hypokalemia. However, recent research has questioned the traditional view of PA as one of the more difficult to control endocrine hypertensions. Instead, it is now thought that PA exists along a broader spectrum of renin-independent aldosterone excess, with classic or overt PA representing just the most severe end. This continuum likely includes a much wider population, even subjects with normal BP who typically wouldn’t undergo RAAS testing. However, these milder forms, often called subclinical PA, have also been linked to inappropriate activation of mineralocorticoid receptors and increased cardiovascular risk [16]. It is logical to assume that these mild and subclinical forms will be discovered more often incidentally, rather than in a targeted screening for PA. Our cases represent typical examples of patients who would not be screened for PA in real clinical practice. Three of them had well-controlled hypertension on conventional antihypertensive treatment, and one patient was even normotensive. This case is of particular interest because the patient had normal BP, body weight, serum potassium levels, and lipid profile. On the other hand, he presented with a well-controlled DM on dual oral antidiabetic combination therapy and bilateral AIs with maximum diameters of 37 mm (left) and 15 mm (right), respectively. Adrenal venous sampling (AVS) was performed, and lateralization was confirmed, indicating excess aldosterone and cortisol production from the larger adenoma. After laparoscopic left adrenalectomy, the patient was still normotensive, with excellent glycemic control on the same antidiabetic therapeutic regimen, but with normalized aldosterone and cortisol levels.

Apart from resistant hypertension, hypokalemia is another hallmark of PA. However, a significant proportion of subjects with PA do not have hypokalemia. In some cases, possible fluctuations in serum potassium levels should be considered, as in our Patient 2. She reported normokalemia from her outpatient laboratory tests, and hypokalemia was found during her admission to the hospital. 

The association between increased aldosterone levels and the risk of overweight and carbohydrate disturbances is still a topic of debate, but should be expected with greater probability in patients with A/C-CoS because of their synergistic action on carbohydrate metabolism. Excess aldosterone-associated hypokalemia decreases both beta-cell insulin secretion and insulin sensitivity at the level of the liver, skeletal muscles, and adipose tissue [17-19]. Cortisol excess is a strong contributor to prediabetic states and overt DM by a complex mechanism. Glucose metabolism disturbances are reported in more than 40% of patients with overt Cushing’s syndrome and in almost 20% of subjects with MACS [20]. Glucocorticoid excess increases gluconeogenesis via stimulation of several key liver enzymes. Another pathogenic mechanism is inhibition of insulin sensitivity in the liver and skeletal muscles, both directly interfering with the insulin receptor signaling pathway and indirectly, via stimulation of lipolysis and the consequent increase of fatty acid circulatory levels. Additionally, glucocorticoid excess exerts a strong inhibitory effect on several steps of the beta cell insulin secretion process [21]. 

We observed carbohydrate disturbances in two of our four patients with mixed aldosterone and cortisol secretion: impaired fasting glucose in Patient 2 and overt DM in Patient 3, both of them with normal BMI. On the contrary, the other two patients were overweight but with normal glucose tolerance. Our case series mirrors the data from the literature. Some researchers have reported an increased risk for overweight, insulin resistance, and/or DM in this subset of subjects with PA [10, 22], whereas other authors have not found a significant difference in the prevalence of diabetes between patients with co-secreting adenomas and those with PA [23]. 

Among our patients with A/C-CoS, three (75%) were diagnosed with dyslipidemia. Published data on the relationship between hyperaldosteronism and lipid profile parameters are inconclusive. Several studies have not observed differences in lipid profiles between PA and subjects with essential hypertension (EH) [24, 25]. Surprisingly, some studies have even shown significantly lower plasma total cholesterol (TC), triglyceride, and low-density lipoprotein cholesterol (LDL-C) levels in patients with PA than in EH [26, 27]. One recently published research study on 267 patients with PA and 267 patients with EH confirmed the lower levels of TC, LDL-C, and non-high-density lipoprotein cholesterol (HDL-C) in PA patients, with a negative correlation between TC, triglyceride, and non-HDL-C levels with plasma aldosterone concentration and ARR. The researchers did not find such a correlation in subjects with EH [28]. Based on these data and the known potential effects of excessive cortisol production in patients with Cushing's syndrome, we can assume that dyslipidemia in patients with co-secretion is mainly due to the cortisol action or genetic factors.

According to the newly developed Expert Consensus on the Primary Aldosteronism Severity Classification (EC-PASC) [29], Patient 1 and Patient 3 exhibited a mild form of PA, Patient 4 a moderate form, and Patient 2 a severe form of the disease.

Surgical treatment should be discussed in all patients with lateralizing PA [15]. Laparoscopic adrenalectomy is the preferred approach, as the tumors are usually small. Laparoscopic surgery was performed on three of the four patients with A/C-CoS in our study. Robotic-assisted surgery was performed on Patient 4, allowing for simultaneous one-stage resection of the right-sided adrenal adenoma and the left-sided renal angiolipoma. Postoperative normalization of the main laboratory parameters (aldosterone, PRA, and cortisol) in all of them was observed. In one case, antihypertensive therapy was discontinued, while in the other two, it was reduced. Complete withdrawal of antihypertensive treatment is not always achievable, especially in cases of delayed diagnosis, when irreversible vascular damage has already occurred. It is also important to consider the additional delay due to the usually long process to definitive diagnosis, which includes screening, confirmatory tests, and subtyping. One recently published international, multicenter retrospective study on 861 patients with PA has demonstrated that the median time-to-adrenalectomy is more than a year (13.5 months, IQR: 6.6-24.5) [30].

None of our patients who underwent surgery has experienced postoperative adrenal insufficiency, as they only had MACS. This can be explained by the fact that the cortisol excess was mild and did not cause significant suppression of the contralateral adrenal gland.

Patient 1 refused surgery, and her AH is still well-controlled on medical therapy not containing MRAs. Despite the good control and normokalemia, low-dose spironolactone should be added to her therapeutic scheme according to the new guidelines [15], which may facilitate the remaining antihypertensive medications and potentially mitigate the aldosterone hypersecretion-associated cardiovascular risk.

Our study has several limitations that should be considered. First, the relatively small number of patients with confirmed mixed aldosterone and cortisol secretion may limit the generalizability of the results. Second, imaging studies were performed on different CT or MRI machines prior to the patients’ admission to the hospital. Third, the varying duration of follow-up among individual patients limited the comprehensive assessment of long-term outcomes.

Nevertheless, our study has its strengths. The findings from this study underscore the need for increased clinical vigilance in detecting these rare cases among all patients with AIs. Limiting the search for mixed aldosterone and cortisol secretion only to patients with PA or overt Cushing’s syndrome would have resulted in missing the milder and subclinical cases, in which early diagnosis and intervention may be more effective. Future studies with larger patient cohorts and standardized protocols are needed to confirm and expand upon the current findings.

## Conclusions

In our cohort of 399 patients with AIs, we identified four cases with mixed A/C-CoS. This corresponds to an approximate prevalence of 1.0%. Mixed aldosterone and cortisol hypersecretion has been usually associated with resistant or uncontrolled hypertension, overweight, and metabolic disturbances (prediabetes, DM, dyslipidemia, etc.). However, in real clinical practice, a wide range of disorders is observed in patients with A/C-CoS, from severe resistant hypertension to rare cases of normotension and from overt metabolic syndrome to a complete absence of metabolic abnormalities. Different presentations or completely asymptomatic (subclinical) forms of A/C-CoS are more likely to be found among patients with AIs. This observation confirms the need for a comprehensive hormonal evaluation of all patients with AIs, regardless of their clinical presentation.
